# Utility of basophil activation testing to assess perioperative anaphylactic reactions in real‐world practice

**DOI:** 10.1002/iid3.175

**Published:** 2017-06-05

**Authors:** Bernadette Eberlein, Sibylle Wigand, Heidrun Lewald, Eberhard Kochs, Johannes Ring, Tilo Biedermann, Ulf Darsow

**Affiliations:** ^1^ Department of Dermatology and Allergy Biederstein Technische Universität München Munich Germany; ^2^ Department of Anesthesiology Klinikum rechts der Isar Technische Universität München Munich Germany

**Keywords:** Basophil activation test, drug allergy, perioperative anaphylaxis

## Abstract

**Introduction:**

Perioperative anaphylactic reactions due to drugs and substances associated with general anesthesia can potentially be life‐threatening. The objective of this study was to investigate the significance of the basophil activation test (BAT) for allergy diagnosis work up.

**Methods:**

A total of 14 patients (5 men, 9 women; mean age: 57.8 years) with clinical records of anaphylactic reactions under general anesthesia were studied by means of anesthesia records, skin and serological tests. Eleven healthy subjects without any history of allergic sensitization to anaesthetic drugs served as controls. BATs based on stimulation of whole blood cells measuring CD63 activation of basophils and using CCR3 as basophil marker by flow cytometry (Flow CAST®, BÜHLMANN Laboratories AG, Schönenbuch, Switzerland) were performed with the following substances (in dependence on the history and the skin tests of the patient): analgesics (acetylsalicylic acid, celecoxib, diclofenac, ibuprofen, indometacin, metamizole, paracetamol, propyphenazone, tramadol), antibiotics (PPL (benzylpenicilloyl polylysine), MDM (minor determinant mixture), amoxicillin, cefuroxime, ciprofloxacin, doxycycline, erythromycin, roxithromycin, sulfamethoxazole, trimethoprim), local anesthetics (articaine, bupivacaine, lidocaine, prilocaine, procaine, methyl‐4‐hydroxybenzoate), narcotics and NMBA (atracurium, cisatracurium, etomidate, neostigmine, midazolam, mivacurium, pancuronium, propofol, pyridostigmine, succinylcholine, sufentanil, thiopental, vecuronium), and other individual substances.

**Results:**

Three patients showed positive results in the BAT: One to metamizole, one to PPL, and one to pancuronium. BATs with these substances were negative in controls.

**Conclusions:**

The BAT should be used complementary to skin tests, especially if IgE‐mediated mechanisms are presumed and skin tests are inconclusive. A positive reaction in BAT identifies the culprit agent with high probability.

## Introduction

Perioperative hypersensitivity reactions can be life‐threatening. The incidence of such reactions is estimated 1 in 13,000 anesthetics up to 1 in 3180 1. Neuromuscular blocking agents (NMBA), antibiotics, induction agents, and opiates are the most common substances, but there is a substantial geographic dfference in the major causes of perioperative anaphylaxis 2. An allergy work‐up to identify the culprit drugs is required to avoid anaphylactic episodes during anesthesia in the future. However, several problems hamper the success in many cases 3.

The history of the reaction is mostly based on anesthesia records which contain ideally the time point of the reaction, the type of reactions, and all given substances, but this is not always documented as it is required. Hidden substances may also cause anaphylaxis. Positive reactions to skin tests with anesthetic agents are below 5% in normal controls at defined concentrations, but exceeding the recommended maximum concentrations can lead to false positive reactions 4. Also for some antibiotics (e.g. ciprofloxacin) irritating concentrations in healthy volunteers are reported. Determination of sIgE is commercially available only for some antibiotics (e.g. amoxicillin, ampicillin, penicilloyl G, penicilloyl V (ImmunoCAP, ThermoFisher, Freiburg, Germany) and has a low sensitivity of less than 40%. Furthermore provocation tests—the gold standard of allergy diagnosis—cannot be performed with NMBA and induction agents.

In addition to these tests the basophil activation test (BAT) can be part of the diagnostic evaluation of drug allergy 5. This cellular in vitro test measures basophil response to allergen cross‐linking IgE on basophil granulocytes. For NMBA, antibiotics and analgesics positive results in the BAT were published 6, 7. It was the aim of this study to investigate the relevance of the BAT for clinical routine allergy diagnosis in patients with perioperative anaphylactic reactions.

## Methods

A total of 14 patients (5 males, 9 females; age: 22–77 years) with a clear history of perioperative anaphylactic reactions recruited consecutively in our allergy unit were included in the study. Two had systemic immediate type reactions of severity grade I, four of severity grade II, four of severity grade III, and four of severity grade IV according to the classification of Ring and Messmer 8. Controls for the BAT comprised 11 individuals (6 males, 5 females, 22–50 years) with a negative history for a perioperative anaphylactic reaction. The study protocol was in accordance with the local ethical committee guidelines and all subjects had given informed consent before being included. Skin prick tests (SPT) and intradermal tests (IDT) were performed. In dependence on the history and the anesthetic protocol the tested drugs included standard blocks of analgesics (acetylsalicylic acid, celecoxib, diclofenac, ibuprofen, indometacin, metamizole, paracetamol, propyphenazone, tramadol), standard blocks of antibiotics (PPL (benzylpenicilloyl polylysine), MDM (minor determinant mixture), amoxicillin, cefuroxime, ciprofloxacin, doxycycline, erythromycin, roxithromycin, sulfamethoxazole, trimethoprim), standard blocks of local anesthetics (articaine, bupivacaine, lidocaine, mepivacaine, prilocaine, procaine, methyl‐4‐hydroxybenzoate), standard blocks of narcotics and NMBA: atracurium, cisatracurium, etomidate, neostigmine, midazolam, mivacurium, pancuronium, propofol, pyridostigmine, succinylcholine, sufentanil, thiopental, vecuronium), and further individual substances, for example, ampicillin, chlorhexidine, clindamycin, dexamethasone, fentanyl, latex, macrogol, metoclopramide, MOVIPREP® (Norgine GmbH, Marburg, Germany), octenisept® (Schülke & Mayr GmbH, Noderstedt, Germany), omeprazole, pantoprazole, patent blue, remifentanil, or rocuronium. SPT were read after 15 min and considered positive when the mean diameter of the wheal and flare reactions was equal or greater than 3 mm.

In patients with negative SPT IDT was done on the ventral aspect of the forearm with part of the above mentioned drugs (antibiotics, local anesthetics, narcotics, NMBA) at lower concentrations (Table 1). IDT were considered to be positive, when the difference to control was greater than 3 mm. Specific IgE‐antibodies against amoxicillin, ampicillin, chlorhexidine, penicillin G, penicillin V, morphine, and latex were perfomed using the system UniCAP 250 (ThermoFisher) in 11 patients. Values of specific IgE below 0.35 kU/L were considered negative.

**Table 1:** Concentrations of the tested standard allergens in skin prick test (SPT) and intradermal test (ID)

Substance	SPT	ID
Analgesics		
Acetyl salicylic acid	Pure in NaCl	n.d.
Celecoxib	Pure in NaCl	n.d.
Diclofenac	Pure in NaCl	n.d.
Ibuprofen	Pure in NaCl	n.d.
Indometacin	Pure in NaCl	n.d.
Metamizole	Pure in NaCl	n.d.
Paracetamol	Pure in NaCl	n.d.
Propyphenazone	Pure in NaCl	n.d.
Tramadol	Pure in NaCl	n.d.
Antibiotics		
PPL (benzylpenicilloyl polylysine)	Undiluted	1:10, undiluted
MDM (minor determinant mixture)	Undiluted	1:100, undiluted
Amoxicillin	Pure in NaCl	0.1%
Cefuroxime	Pure in NaCl	0.1%
Ciprofloxacin	Pure in NaCl	n.d.
Doxycycline	Pure in NaCl	n.d.
Erythromycin	Pure in NaCl	n.d.
Roxithromycin	Pure in NaCl	n.d.
Sulfamethoxazole/Trimethoprim	Pure in NaCl	n.d.
Local anesthetics		
Articaine	2%	0.1%
Bupivacaine	0.5%	0.1%
Lidocaine	2%	0.1%
Mepivacaine	2%	0.1%
Prilocaine	2%	0.1%
Procaine	2%	0.1%
Methyl‐4‐hydroxybenzoate	0.1%	0.1%
Narcotics and NMBA		
Atracurium 10 mg/mL	Undiluted	1:100
Cicatracurium 2 mg/mL	Undiluted	1:100
Etomidate 2 mg/mL	Undiluted	1:10
Neostigmine 0.5 mg/mL	Undiluted	1:100
Midazolam 5 mg/mL	Undiluted	0:10
Mivacurium 2 mg/mL	Undiluted	1:1000
Pancouronium 2 mg/mL	Undiluted	1:10
Propofol 10 mg/mL	Undiluted	1:10
Pyridostigmine 5 mg/mL	Undiluted	1:100
Succinylcholine 2 mg/mL	Undiluted	1:100
Sufentanil 0.005 mg/mL	Undiluted	1:10
Thiopental 25 mg/mL	Undiluted	1:100
Vecuronium 2 mg/mL	Undiluted	1:10

n.d., not done.

The Flow2 CAST® (BÜHLMANN Laboratories AG, Schönenbuch, Switzerland) was performed as previously described 9 with the following substances (in dependence on the history and the results of the skin tests): Analgesics (fentanyl, metamizole, paracetamol, piritramide, remifentanil, sufentanil, tramadol), antibiotics (ampicillin, cefazolin, cefuroxime, ciprofloxacin, clindamycin, PPL, MDM), local anesthetics (bupivacaine, lidocaine, mepivacaine, ropivacaine), narcotics (etomidate, midazolam, propofol, pyridostigmine, thiopental), NMBA (atracurium, cisatracurium, mivacurium, neuromuscular blocking mix, pancuronium, rocuronium, suxamethonium, vecuronium), and others (dexamethasone, hydroxyethyl starch [HES], patent blue, suprarenin). Concentrations used are listed in Table 2. In order to consider the results as positive a cut‐off ≥5 % activated basophils and a stimulation index ≥2 (SI = allergen stimulation divided by negative control) was used.

**Table 2:** Concentrations (after reconstitution) of the tested allergens in BAT. For concentrations in stimulation values have to be divided by 4.4

Substance	Concentration 1	Concentration 2
Analgesics		
Fentanyl^a^	500–1040 ng/mL^c^	250–500 ng/mL^c^
Metamizole^b^	50 μg/mL	10 μg/mL
Paracetamol^b^	10 μg/mL	2 μg/mL
Piritramide^a^	5.5 μg/mL	1.1 μg/mL
Remifentanil^a^	60 ng/mL	30 ng/mL
Sufentanil^a^	440–640 ng/mL^c^	220–320 ng/mL^c^
Tramadol^a^	100 mg/mL	20 mg/mL
Antibiotics		
Ampicillin^b^	10 mg/mL	2 mg/mL
Cefazolin^b^	2.5 mg/mL	0.5 mg/mL
Cefuroxime^b^	2.5 mg/mL	0.5 mg/mL
Ciprofloxacin^b^	100 μg/mL	20 μg/mL
Clindamycin^a^	220 μg/mL	110 μg/mL
PPL^b^	50 μg/mL	10 μg/mL
MDM^b^	1 mg/mL	0.2 mg/mL
Local anesthetics		
Bupivacaine^b^	400 μg/mL	80 μg/mL
Lidocaine^b^	250 μg/mL	50 μg/mL
Mepivacaine^b^	400 μg/mL	80 μg/mL
Ropivacaine^a^	800 μg/mL	400 μg/mL
Narcotics		
Etomidate^a^	2.94 μg/mL	1.47 μg/mL
Midazolam^a^	11 ng/mL	5.5 ng/mL
Propofol^b^	1 mg/mL	0.2 mg/mL
Pyridostigmine^a^	22 μg/mL	11 μg/mL
Thiopental^a^	0.38–0.44 mg/mL^c^	0.19–0.22 mg/mL^c^
NMBA		
Atracurium^b^	5 μg/mL	1 μg/mL
Cisatracurium^b^	400 μg/mL	80 μg/mL
Mivacurium^b^	1 mg/mL	0.2 mg/mL
Neuromuscular blocking mix^b^	4.3 mg/mL	0.86 mg/mL
Pancuronium^b^	1 mg/mL	0.2 mg/mL
Rocuronium^b^	1 mg/mL	0.2 mg/mL
Suxamethonium^b^	10 mg/mL	2 mg/mL
Vecuronium^b^	250 μg/mL	50 μg/mL
Others		
Dexamethasone^a^	5.86 μg/mL	2.93 μg/mL
Hydroxyethyl starch^a^	60 μg/mL	12 μg/mL
Patent blue V^b^	250 μg/mL	50 μg/mL
Suprarenin^a^	0.146 μg/mL	0.073 μg/mL

^a^ Commercially available medication.

^b^ Purchased from BÜHLMANN Laboratories AG, Schönenbuch, Switzerland.

^c^ Concentration depending on body weight of the patient.

## Results

Analgesics were used in history in four patients with three patients showing positive results in the SPT to metamizole and one patient to tramadol. Antibiotics were given in history in nine patients. Out of the 14 patients tested six cases showed positive results in the SPT to amoxicillin, ampicillin, cefuroxime, ciproflocaxin, clindamycin, doxycycline, or PPL and two cases in the IDT to cefuroxime or MDM. One patient had sIgE antibodies to betalactams (CAP class 3 to amoxicillin, ampicillin, penicilloyl G, penicilloyl V). Local anesthetics were given in history in five patients, none of them had a positive skin test reaction. Narcotics were used in history in all cases. One patient had a positive SPT to propofol and 11 patients positive IDTs to etomidate, midazolam, pyridostigmine, succinylcholine, or thiopental. NMBA were used in history in eight patients. Out of the 14 patients six cases showed positive results in the SPT and 10 cases in the IDT to atracurium, cisatracurium, mivacurium, pancuronium, succinylcholine, or vecuronium. SIgE to morphine was negative in all cases. Other substances involved were SPT positive in two cases (patent blue and latex).

Three patients showed positive results in the BAT: One patient revealed a basophil activation of 49.1% to metamizole (SI = Stimulation Index: 114.2) at a concentration (after reconstitution) of 50 μg/mL, another patient showed a basophil activation of 7.8% (SI 39.2), and 22.1% to PPL (SI 110.9) at two different concentrations (50 and 10 μg/mL after reconstitution), respectively and one patient revealed an activation of 7.8% (SI 11.2) to pancuronium at a concentration of 0.2 mg/mL after reconstitution. BATs with these substances were negative in controls. Comparisons of the positive results in the different tests are listed in Table 3.

**Table 3:** Number of patients with the substance group used in anesthesia, number of positive reactions in skin tests, determination of specific IgE and basophil activation tests (Flow2 CAST®) in the study group (*n* = 14)

	Analgesics	Antibiotics	Local anesthetics	Narcotics	NMBA
History	4	9	5	14	8
Prick test	4	6	0	1	6
Intradermal test	n.d.	2	0	11	10
sIgE	n.a.	1	n.a.	n.a.	n.a.
Basophil activation test	1	1	0	1	0

n.a., not applicable; n.d., not done.

## Discussion

This study shows a discrepancy between the number of positive reactions in skin testing and number of positive results in BAT. This might be due to different reasons: On the one hand non‐IgE mediated mechanisms or irritative reactions of drugs (especially of narcotics and NMBA in the intracutaneous test) may lead to positive skin test results which cannot be recovered in vitro. This is supported by the negative results in IgE morphine determination being a very sensitive biomarker for quaterny ammonium sensitization in case of suspected hypersensitivity to NMBA 10. On the other hand IgE‐mediated reactions which were found for betalactams and metamizole in the BAT in two cases seem to be less frequent in perioperative reactions. Furthermore sensitivity for betalactams and metamizole in the BAT is about 40–50% 9, 11. Therefore the culprit agent would have not been detected by BAT in 50–60% of the cases. As specificity for both drugs is high (80% fo betalactams, 100% to metamizole), a positive BAT points out the relevant substance. This was underlined in our two cases by a positive skin test to amoxicillin, ampicillin, PPL and sIgE to amoxicillin, ampicillin, penicilloyl B, and penicilloyl V in the one case (eliciting drug ampicillin) and a convincing history (two anaphylactic reactions in which metamizole was involved) as well as a positive skin test to metamizole in the other case. In the third case pancuronium (not atracurium, cisatracurium, mivacurium or vencuronium) showed a positive result in the BAT, but the NMBA given during anesthesia was cisatracurium and the intracutaneous test was positive to atracurium, mivacurium, and pancuronium. Cross‐reactivity among these substances can be assumed. For NMBA it was shown that skin test and BAT have an excellent negative predictive value especially if rocuronium was involved 6.

The BAT should be used complementary to skin tests in patients with perioperative anaphylactoid reactions, especially if IgE‐mediated mechanisms are presumed and skin tests are inconclusive. Due to a good specificity a positive reaction with a high activation in BAT identifies the culprit agent in perioperative hypersensitivity reactions with high probability. This is advantageous in cases where a provocation test cannot be performed due to the drug effect (e.g. in NMBA). In other cases drug challenges that are time consuming, expensive and risky with regard to severe allergic reactions can be deferred or avoided (e.g. in case of urgent treatments under anesthesia). Furthermore BAT is also applicable in patients with contraindications for skin or provocation tests (e.g. intake of β‐blockers or ACE inhibitors).

## Conflicts of Interest

None declared.
